# An Atlas of Ophthalmoscopy

**Published:** 1896-07

**Authors:** 


An Atlas of Ophthalmoscopy. With an Introduction to the Use of the Ophthalmoscope. By Dr. O. Haab, Professor of Ophthalmology, University of Zurich. Translated and edited by Ernest Clarke, M. D., B. S. (Lond.), Fellow of the Royal College of Surgeons; Surgeon to the Central London Ophthalmic Hospital; Ophthalmic Surgeon to the Miller Hospital, etc. Duodecimo. With wood-cuts and sixty-four colored plates. Price, $3.50. New York: William Wood & Co. 1895.

This atlas is preëminently a work for the student of ophthalmoscopy. In the introduction is given brief instruction in the use of the ophthalmoscope, embodying an explanation of the instrument; how to use it by both the direct and indirect methods; the measurement of errors of refraction; the determination of the size of the ophthalmoscopic image and of inequalities in the fundus; the application of the shadow test; the method of conducting an ophthalmoscopic examination, and the ophthalmoscopic appearance of the normal fundus. These subjects are treated very concisely, but clearly, and in a manner suited to the needs of a beginner.

Following the introduction is a series of well-executed colored plates, sixty-four in number, containing eighty-six figures illustrating the more common appearances seen in healthy and diseased fundi. The various aspects of the normal fundus are shown by seven figures; of congenital malformations, by eight; of diseases of the optic nerve, by fourteen; of diseases of the retina, by forty-one; of diseases of the choroid, by sixteen. Facing each plate is a description of it, which greatly facilitates its study. The volume is in a most convenient size and can easily be carried to the clinic room in the pocket.

The convenient size, the subject matter, the well-drawn plates, and the large number of fundus pictures portrayed, render this volume of superior value to the student. The translator, Mr. Clarke, of London, and the publishers have done the profession great service in putting in English dress this excellent work of Dr. Haab. A. A. H.

